# Edaravone acts as a potential therapeutic drug against pentylenetetrazole‐induced epilepsy in male albino rats by downregulating cyclooxygenase‐II

**DOI:** 10.1002/brb3.1156

**Published:** 2018-12-01

**Authors:** Liang‐min Liu, Ning Wang, Yan Lu, Wei‐ping Wang

**Affiliations:** ^1^ Key Laboratory of Neurology of Hebei Province The Second Hospital of Hebei Medical University Shijiazhuang Hebei PR China; ^2^ Department of Pediatric Intensive Care Unit Anyang Traditional Chinese Medicine Hospital Anyang Henan PR China

**Keywords:** antioxidant, apoptosis, cyclooxygenase, edaravone, rats

## Abstract

**Introduction:**

The effects of edaravone against pentylenetetrazole (PTZ)‐induced epilepsy in male albino rats were investigated. Edaravone is a well‐known commercial drug used in the treatment of strokes and amyotrophic lateral sclerosis (ALS). Antioxidant and free radical scavenging activities of edaravone have been reported in patients with ALS.

**Methods:**

In this study, the experimental groups were as follows: sham, control, 5 mg/kg edaravone, and 10 mg/kg edaravone. Behavioral assessment, determination of biochemical markers, apoptosis, nitric oxide (NO), and mRNA and protein expression of cyclooxygenase‐II (COX‐II) were carried out. Seizure incidence, including generalized tonic–clonic seizure (GTCS) and minimal clonic seizure (MCS), was directly associated with PTZ administration in rats.

**Results:**

Edaravone supplementation substantially increased MCS and GTCS latency in rats, and biochemical markers were significantly altered in the brain tissue of PTZ‐treated rats. Edaravone treatment normalized altered biochemical markers compared with the untreated control. Apoptosis and NO levels were significantly reduced by more than 50% compared to their respective controls. COX‐II mRNA was increased by 130% in PTZ‐treated rats, while edaravone supplementation reduced mRNA and protein expression of COX‐II by more than 20% and 40%, respectively. Immunohistochemistry indicated that COX‐II protein expression was reduced by 13.2% and 33.7% following supplementation with 5 and 10 mg/kg edaravone, respectively.

**Conclusion:**

Taken together, our results suggest that edaravone functions by downregulating the levels of COX‐II and NO and is a potential candidate for the treatment of PTZ‐induced epilepsy.

## 1 INTRODUCTION

Edaravone is a well‐known commercial drug used in the treatment of strokes and amyotrophic lateral sclerosis (ALS) (Miyaji et al., 2015). Antioxidant and free radical scavenging activities of edaravone have been reported in patients with ALS (Petrov, Mansfield, Moussy, & Hermine, 2017). Researchers have reported that edaravone exhibits a neuronal protective effect by reducing brain damage in a model of acute ischemia (Watanabe, Tanaka, Watanabe, Takamatsu, & Tobe, 2004). Edaravone has been reported to be active against lipid peroxidation and to exhibit free radical quenching activity (Watanabe, Yuki, Egawa, & Nishi, 1994; Yoshida, Yanai, Namiki, & Fukatsu‐Sasaki, 2006). Cheng and Zhang (2014) reported that edaravone treatment reduced apoptosis in the hippocampus following pentylenetetrazole (PTZ)‐induced seizures.

Cyclooxygenases (COXs) are enzymes involved in the conversion of arachidonic acid to prostaglandins, which play a major role in the inflammatory reaction (Simmons, Botting, & Hla, 2004). Cyclooxygenases exist in two forms, COX‐I and COX‐II, in different cell types. COX‐II is predominantly found in the central nervous system (CNS) and is induced in areas of inflammation following injury (Choi, Aid, & Bosetti, 2009). Choi et al. (2009) reported that COX‐II is primarily expressed in the hippocampus and cerebral cortex and is involved in seizure onset. Chen, Magee, and Bazan (2002) reported that COX‐II regulates prolonged synaptic plasticity and cell membrane excitability, indicating the importance of COX‐II in convulsion. Nitric oxide (NO) is a well‐known neurotransmitter produced from arginine by nitric oxide synthase (NOS; Zhou & Zhu, 2009). NO is known to increase PTZ‐induced seizures (Riazi et al., 2006). The simultaneous synthesis of prostaglandins and NO has been reported in various tissues, as well as their putative roles in inflammatory reactions, indicating a possible interaction between prostaglandins and NO (Mollace, Muscoli, Masini, Cuzzocrea, & Salvemini, 2005). To date, no study has investigated the effects of edaravone on the levels of COX‐II and NO in a rat PTZ‐induced seizure model.

## 2 MATERIALS AND METHODS

Twenty‐four male rats (Albino, Wistar strain, 200–220 g) were obtained from the animal facility of the Key Laboratory of Neurology of Hebei Province, the Second Hospital of Hebei Medical University (Shijiazhuang, Hebei, PR China). The rats were distributed into four homogeneous groups, with six rats in each group. Water and food were provided ad libitum, and rats were kept in cages with standard light and dark period. All experimental procedures involving rats were monitored and approved by the Second Hospital of Hebei Medical University (Shijiazhuang, Hebei, PR China).

### 2.1 Treatment

The experimental groups were as follows: sham, control, 5 mg/kg edaravone, and 10 mg/kg edaravone. PTZ (Sigma‐Aldrich Inc., China) was dissolved in normal saline (0.9%) and administered intraperitoneally (Ebrahimzadeh Bideskan et al., 2011). PTZ (90 mg/kg body weight) was administered to rats in control, 5 mg/kg edaravone, and 10 mg/kg edaravone groups. Saline was given to the sham and control groups instead of edaravone. Doses were administered orally for 15 consecutive days.

### 2.2 Behavioral assessment

Following administration of PTZ, rats were kept in a Plexiglas chamber (30 × 30 × 30 cm) and observed for 1 hr. Generalized tonic–clonic seizure (GTCS) and latency to the first minimal clonic seizure (MCS), the incidence of GTCS and MCS, and mortality rate were calculated as criteria for the behavioral response to PTZ administration (Ebrahimzadeh Bideskan et al., 2011; Hosseini, Sadeghnia, Salehabadi, Alavi, & Gorji, 2009).

### 2.3 Mortality rate

Higher mortality rate was observed in control rats (70%–80%). The administration of edaravone (5 mg/kg) slightly reduced mortality rate (15%), while administration of edaravone (10 mg/kg) significantly reduced (27%) compared to their respective controls (data not shown).

### 2.4 Biochemical markers

Catalase activity was determined by the addition of 500 µl of phosphate buffer, 500 µl of tissue homogenate, 500 µl of H_2_O_2_, and 500 µl of TiOSO_4_ to the reaction tube. The absorbance was measured at 420 nm (Feuers, Weindruch, Leakey, Duffy, & Hart, 1997). Superoxide dismutase (SOD) activity was determined by the addition of 0.1 ml of tissue homogenate, 1.2 ml of sodium phosphate buffer, 0.3 ml of nitro blue tetrazolium, and 0.2 ml of NADH. The absorbance was measured at 560 nm (Feuers et al., 1997). Glutathione peroxidase (Gpx) activity in tissue homogenate was determined by measuring the absorbance at 340 nm (Baydas et al., 2002). Reduced glutathione (GSH) levels in the tissue homogenate were determined based on Ellman's reaction. The final product was measured by the absorbance at 412 nm (Kaddour et al., 2016). The malondialdehyde (MDA) content was measured as an index of lipid peroxidation in the tissue homogenate by measuring thiobarbituric acid reactive species (TBARS). The resultant final product was measured the absorbance at 534 nm (Power & Blumbergs, 2009). Total thiol content in the tissue homogenate was determined according to the previously reported method. Briefly, tissue homogenate and colorimetric probe were added to the 96‐well plates. The probe reacts with sulfhydryl to release a chromophore, and final the absorbance was measured at 450 nm (Khodabandehloo et al., 2013).

### 2.5 Apoptosis assay

Terminal deoxynucleotidyl transferase dUTP nick end labeling (TUNEL) and staining were performed in hippocampal sections as previously described. Hippocampal tissues were excised and perfused in normal saline, and then, hippocampal tissues were fixed in 10% neutral formalin (10%) for 24 hr. Hippocampal sections were dehydrated by graded alcohol and embedded in paraffin film. Then, sections were cut into (4–5 µm) thickness by a rotary microtome. Then, terminal deoxynucleotidyl transferase dUTP nick end label (TUNEL) staining was performed. An optimal signal‐to‐background ratio was obtained at 1:4 dilution of the antibody. Then, reaction with 0.05% of 3–3′‐diaminobenzidine tetrahydrochloride (DAB) was monitored under the microscope (Graziano, Spoon, Cockrell, Rowse, & Gonzales, 2001).

### 2.6 Determination of NO levels

Nitric oxide (NO) level was determined as in Shaheen et al. (2016). Nitrate and nitrite are final products of NO metabolism and these levels used for the quantification of NO. Sum of nitrate and nitrite accounts for the total NO concentration. Griess's reaction was used for the determination of NO level in serum based on the nitrite concentration. In the serum, nitrate was reduced nitrite in the presence of cadmium (Sigma‐Aldrich Inc., Shanghai, China). Then, nitrite was converted to nitric acid that reacts with Griess's reagent (Sigma‐Aldrich Inc., Shanghai, China) to produce color. Serum level of nitrite was determined by measuring absorbance at 540 nm in a spectrophotometer.

### 2.7 Real‐time polymerase chain reaction (RT‐PCR)

Total RNA was isolated from hippocampal tissue homogenate and converted into cDNA using oligo (dT) primers. The cDNA was used for qRT‐PCR using primers specific for COX‐II (forward: 5′‐TGCGATGCTCTTCCGAGCTGTGCT‐3′, reverse: 5′‐TCAGGAAGTTCCTTATTTCCTTTC‐3′) and β‐actin (forward: 5′‐GATGGCCACGGCTGCTTC‐3′, reverse: 5′‐TGCCTCAGGGCAGCGGAA‐3′) as the internal control. Relative expression ratios were determined according to Langnaese, John, Schweizer, Ebmeyer, and Keilhoff (2008).

### 2.8 Western blot analysis

Proteins in the hippocampal tissue homogenates were separated by sodium dodecyl sulfate‐polyacrylamide gel electrophoresis, transferred to polyvinylidene difluoride membranes, and incubated with the anti‐COX‐II primary antibody (ab15191; Abcam) for 12 hr, followed by the horseradish peroxidase‐conjugated goat anti‐rabbit IgG secondary antibody (A0545‐1ML; Sigma‐Aldrich) for 1 hr. Enhanced chemiluminescence was used to determine protein levels of COX‐II (Nie et al., 2017).

### 2.9 Immunohistochemistry

Hippocampal tissues were excised and perfused in normal saline, and then, hippocampal tissues were fixed in 10% neutral formalin (10%) for 24 hr. Hippocampal sections were dehydrated by graded alcohol and embedded in paraffin film. Paraffin‐embedded hippocampal sections were dewaxed, washed with deionized water, and rinsed with PBS. Hippocampal sections were incubated with goat serum (0.5% bovine serum albumin and 0.1% Tween) for 30 min. Then, sections were incubated with the anti‐COX‐II primary antibody (ab15191; Abcam) at 4°C for 12 hr, followed by the goat anti‐mouse secondary antibody (ab205719; Abcam). Then, sections were counterstained with hematoxylin for 60 s. Finally, sections were imaged, and expressions were quantified (Rehg, Bush, & Ward, 2012).

### 2.10 Statistical analysis

Values are given as means with *SD*. One‐way ANOVA (SPSS 17, IBM SPSS Modeler, Hong Kong) was applied for statistical analyses of data, and Tukey's post hoc tests were used for multiple comparisons. *p*‐values <0.05 were considered statistically significant.

## 3 RESULTS

**Figure 1:**
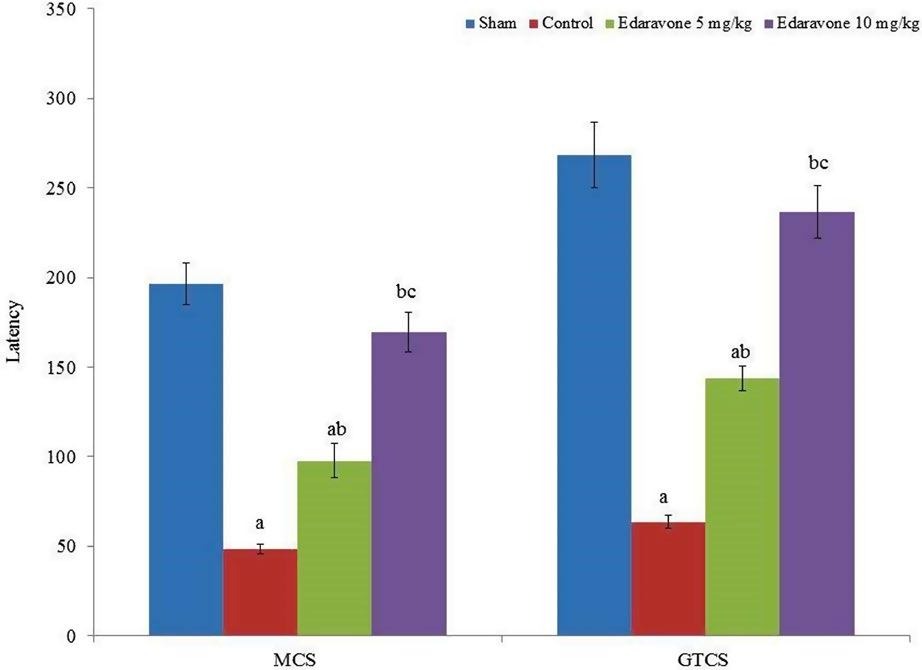
Effect of edaravone on generalized tonic–clonic seizure and latency to the first minimal clonic seizure in a pentylenetetrazole (PTZ)‐induced seizure rat model. Results are presented as mean with standard error of the mean (*SEM*). ^a^ *p* < 0.05 versus sham, ^b^ *p* < 0.05 versus control, and ^c^ *p* < 0.05 versus 5 mg/kg edaravone

In this study, the protective effects of edaravone on PTZ‐induced epilepsy in male albino rats were investigated. Seizure incidence, including GTCS and MCS, was directly associated with PTZ administration in rats. Edaravone supplementation substantially increased MCS and GTCS latency in the rats (Figure 1, *p* < 0.05). Biochemical markers were significantly altered in the brain tissue of PTZ‐treated rats. Lipid peroxidation, SOD and catalase activities, GSH and Gpx levels, and total thiol content were altered considerably in PTZ rats compared to the sham group (Table 1, *p* < 0.05). Administration of PTZ significantly increased lipid peroxidation, whereas SOD and catalase activities, GSH and Gpx levels, and total thiol content were significantly reduced in the control compared to the sham group (Table 1, *p* < 0.05).

**Table 1:** Effect of edaravone on lipid peroxidation, antioxidant markers, and thiol content in pentylenetetrazole‐induced epilepsy in male albino rats

Biochemical markers	Sham	Control	Edaravone 5 mg/kg	Edaravone 10 mg/kg
SOD (U/ml)	296.8 ± 18.4	116 ± 9.1^a^	188.6 ± 13.6a,b	267 ± 16.4b,c
Catalase (U/ml)	8.6 ± 0.4	3.1 ± 0.19^a^	5.1 ± 0.22a,b	7.6 ± 0.25b,c
Gpx (U/ml)	0.44 ± 0.05	0.13 ± 0.002^a^	0.23 ± 0.005a,b	0.37 ± 0.006b,c
GSH (nmol/ml)	0.55 ± 0.04	0.16 ± 0.005^a^	0.27 ± 0.02a,b	0.45 ± 0.006b,c
MDA (nmol/ml)	0.4 ± 0.02	1.2 ± 0.05^a^	0.85 ± 0.05a,b	0.53 ± 0.04b,c
Thiol content (mmol/L)	26.4 ± 1.2	6.9 ± 0.16^a^	12.3 ± 0.9a,b	23.6 ± 1.5b,c

^a^ *p *< 0.05 versus sham. ^b^ *p *< 0.05 versus control. ^c^ *p *< 0.05 versus edaravone 5 mg/kg.

Pentylenetetrazole administration affected brain hippocampal tissue by increasing lipid peroxidation and decreasing SOD and catalase activities, GSH and Gpx levels, and total thiol content (Table 1, *p* < 0.05). Edaravone supplementation increased SOD and catalase activities by more than 40% and increased Gpx activity and GSH content by more than 40%. Malondialdehyde levels were reduced by 0.85 and 0.53 nmol/g in the 5 and 10 mg/kg edaravone groups, respectively (Table 1, *p* < 0.05). Total thiol content was drastically reduced in PTZ‐treated rats compared to the sham group. However, edaravone supplementation increased thiol content by more than 50% compared to the control group (Table 1, *p* < 0.05).

**Figure 2:**
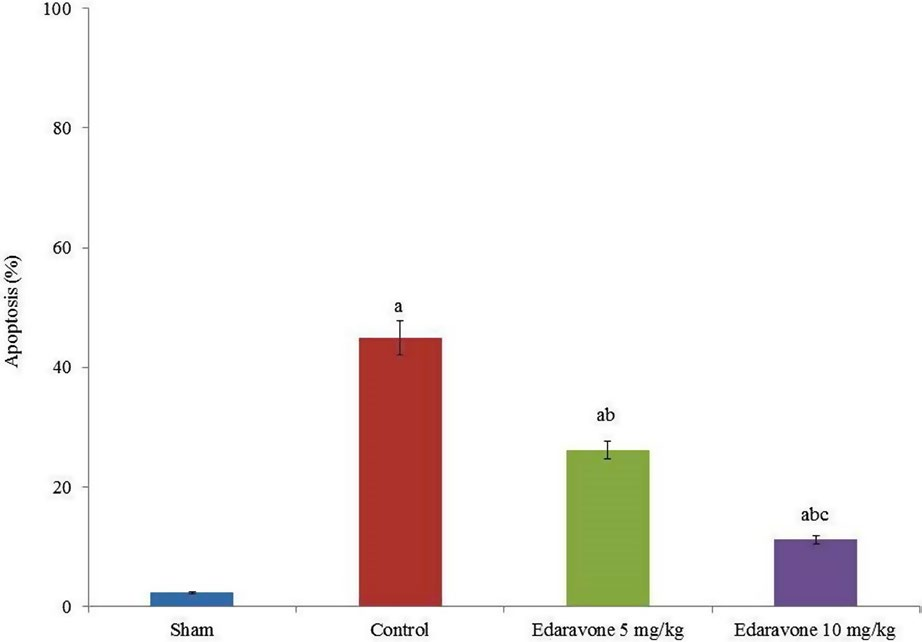
Effect of edaravone on apoptosis in a PTZ‐induced seizure rat model. Results are presented as mean with *SEM*. ^a^ *p* < 0.05 versus sham, ^b^ *p* < 0.05 versus control, and ^c^ *p* < 0.05 versus 5 mg/kg edaravone

**Figure 3:**
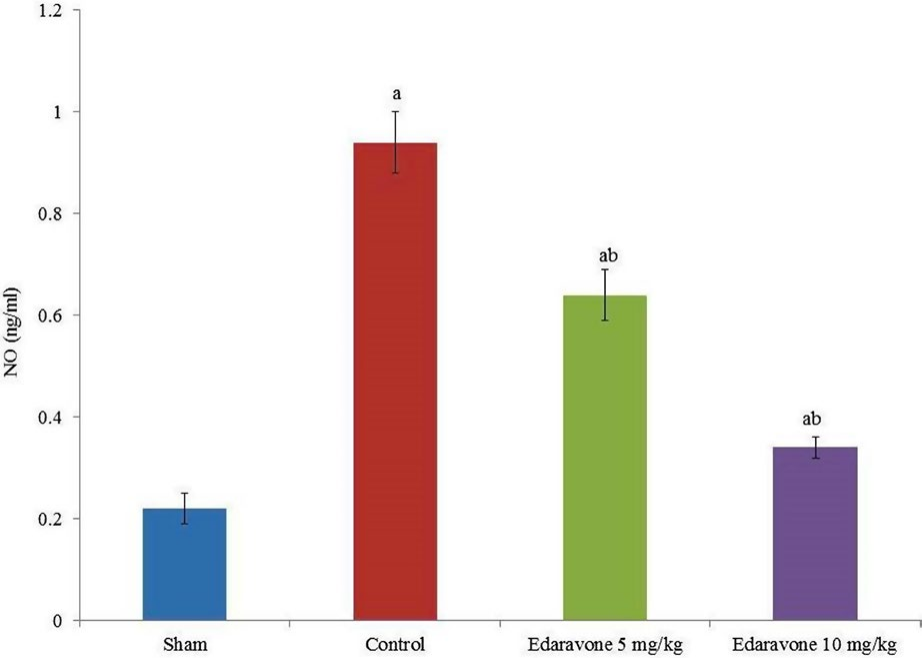
Effect of edaravone on nitric oxide in a PTZ‐induced seizure rat model. Results are presented as mean with *SEM*. ^a^ *p* < 0.05 versus sham, ^b^ *p* < 0.05 versus control, and ^c^ *p* < 0.05 versus 5 mg/kg edaravone

**Figure 4:**
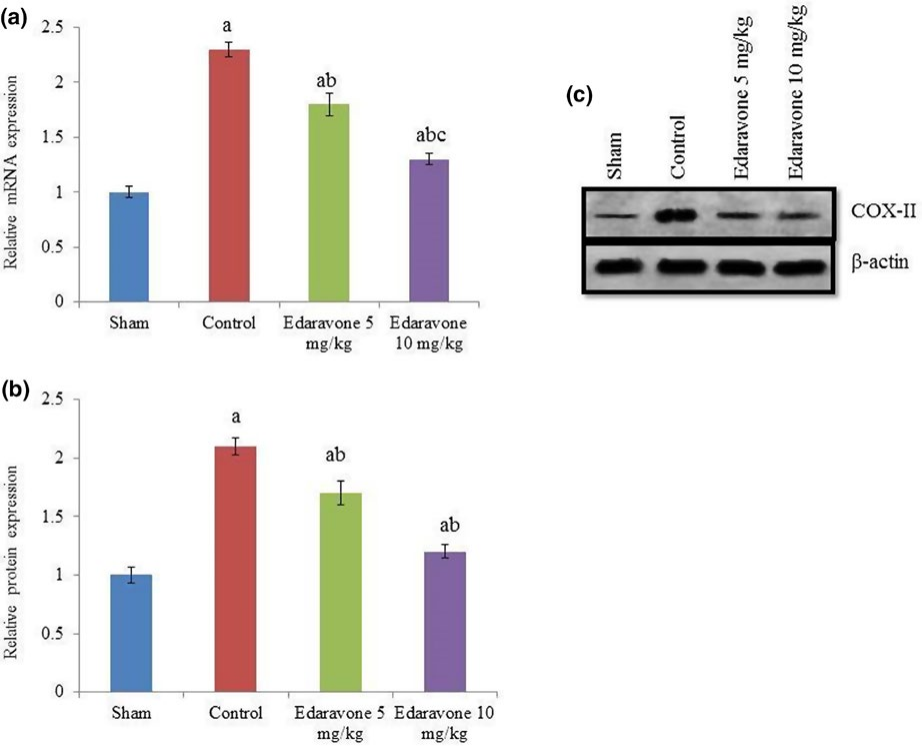
Effect of edaravone on cyclooxygenase‐II (COX‐II) expression in a PTZ‐induced seizure rat model. (a) mRNA expression analysis of COX‐II. (b) Western blotting of COX‐II. (c) Densitometry analysis of Western blotting of COX‐II. Results are presented as mean with *SEM*. ^a^ *p* < 0.05 versus sham, ^b^ *p* < 0.05 versus control, and ^c^ *p* < 0.05 versus 5 mg/kg edaravone

**Figure 5:**
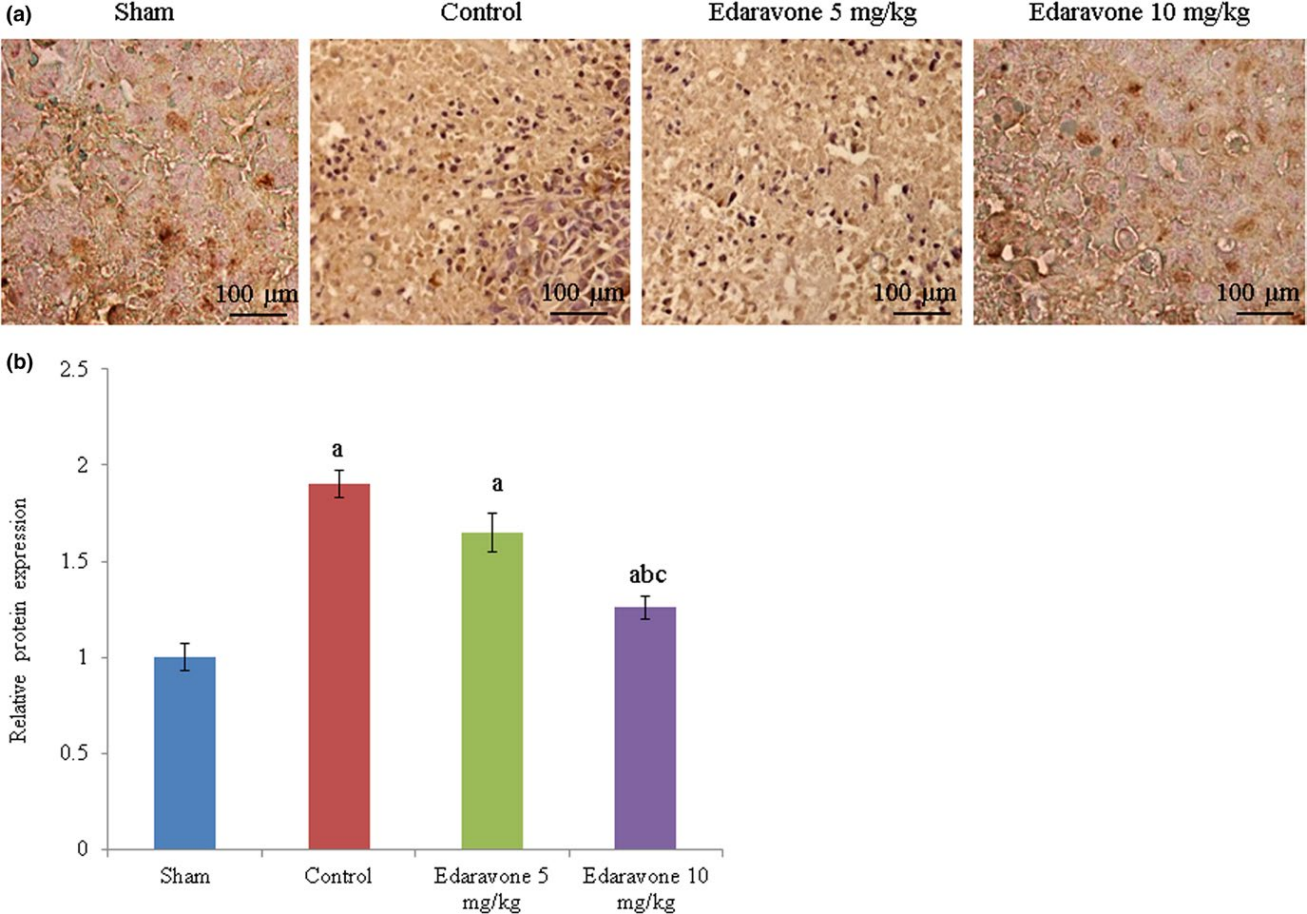
Effect of edaravone on COX‐II expression in a PTZ‐induced seizure rat model. (a) Immunohistochemical analysis of COX‐II protein expression. (b) Quantitative analysis of immunohistochemical images of COX‐II. Results are presented as mean with *SEM*. ^a^ *p* < 0.05 versus sham, ^b^ *p* < 0.05 versus control, and ^c^ *p* < 0.05 versus 5 mg/kg edaravone. Magnification, 40×. Scale bar = 100 µm

Apoptosis was drastically increased in PTZ‐treated rats compared to the sham group. However, edaravone supplementation reduced apoptosis by more than 50% (Figure 2, *p* < 0.05). The NO levels were drastically increased in PTZ‐treated rats compared to the sham group. However, the NO level was reduced by 31.9% and 63.8% following supplementation with 5 and 10 mg/kg edaravone, respectively (Figure 3, *p* < 0.05). COX‐II mRNA expression was increased by 130% in PTZ‐treated rats compared with the sham group. However, the COX‐II mRNA level was reduced by 21.7% and 43.5% following supplementation with 5 and 10 mg/kg edaravone, respectively (Figure 4a, *p* < 0.05), while COX‐II protein expression was reduced by 19% and 42.8% following supplementation with 5 and 10 mg/kg edaravone, respectively (Figure 4c, *p* < 0.05). This was further confirmed by immunohistochemical analysis of COX‐II protein, which was reduced by 13.2% and 33.7% following supplementation with 5 and 10 mg/kg edaravone, respectively (Figure 5, *p* < 0.05).

## 4 DISCUSSION

In this study, the protective effects of edaravone on PTZ‐induced epilepsy in male albino rats were investigated. Our experimental results confirmed that edaravone inhibits PTZ‐induced seizures. The mechanism of PTZ‐induced seizure is not well understood. Researchers have reported that the active role of picrotoxin binding site on the GABA‐A receptor complex. Diazepam and phenobarbital are act as anti‐convulsant agent and are well‐known ligands of the GABA‐A receptor complex. It is believed that PTZ has opposite effect when binds to the GABA‐A receptor complex (Squires, Saederup, Crawley, Skolnick, & Paul, 1984). Antioxidant and free radical scavenging activities of edaravone have been reported in patients with ALS (Petrov et al., 2017). Edaravone has also been reported to exhibit a neuronal protective effect by reducing brain damage in a model of acute ischemia (Watanabe et al., 2004). Additionally, edaravone has been shown to be active against lipid peroxidation and exhibits free radical quenching activity (Watanabe et al., 1994; Yoshida et al., 2006).

Yang and Chen (2008) have demonstrated that COX‐II synthesizes prostaglandins following stimuli from seizures, inflammatory reactions, and brain trauma, and Choi et al. (2009) have shown that COX‐II is expressed in the CNS, hippocampus, and cortex. Our investigation indicates that edaravone can decrease PTZ‐induced seizures by downregulating COX‐II. Yang and Chen (2008) reported that prostaglandin E2 and COX‐II together could regulate membrane excitability. Several studies (Gholipour et al., 2008; Toscano, Kingsley, Marnett, & Bosetti, 2008) have also shown that the effects of COX‐II overexpression can be reduced via the *N*‐methyl‐D‐aspartate receptor (NMDA) receptor inhibition.

Strauss and Marini (2002) have demonstrated that COX‐II inhibitors inhibit neurotoxicity. Thus, it is possible that edaravone acts as a COX‐II inhibitor and thus may function as an anti‐convulsant agent. Feil and Kleppisch (2008) have shown that NO acts as a neurotransmitter responsible for various pathological and physiological functions. NO plays a critical role in cycline guanine monophosphate formation and further leads to the formation of NMDA receptors in the CNS.

Itoh et al. (2004) reported increased levels of NOS in the neurons of a PTZ‐induced seizure model. Arancio et al. (2001) reported that NO is expressed in hippocampal culture and augmented synaptic transmission. Therefore, increased levels of NO are believed to participate in the pathophysiology of epilepsy. Several studies have reported that COX‐II and NO can interact in cardiomyopathy, osteoarthritis, renal perfusion, and angiogenesis (Beierwaltes, 2002; Davel et al., 2002). Finally, Hughes et al. (1999) demonstrated that NO upregulates COX‐II expression. However, to the best of our knowledge, the crosstalk between prostaglandins and NO has not yet been investigated.

## 5 CONCLUSIONS

Edaravone supplementation significantly normalized altered lipid peroxidation and antioxidant biochemical markers. Apoptosis and NO levels were reduced compared to their respective controls. mRNA and protein expression of COX‐II was substantially reduced following edaravone supplementation. Taken together, our results suggest that edaravone is a potential candidate for the treatment of PTZ‐induced epilepsy and functions by downregulating the levels of COX‐II and NO.

## CONFLICT OF INTEREST

Authors declare that they have no conflict of interest.
